# Multidisciplinary Approach in Aesthetic Smile Design: A Clinical Case Report

**DOI:** 10.7759/cureus.90912

**Published:** 2025-08-24

**Authors:** Darren Tun Yi Ong, Melwin Mathew, Li Jia Ong, Kai Jing Lee

**Affiliations:** 1 Dentistry, Manipal University College Malaysia, Melaka, MYS; 2 Dentistry/Periodontics, Beresford Dental Clinic, London, GBR

**Keywords:** aesthetic restorations, coronally advanced flap, crown, direct veneer, multidisciplinary

## Abstract

An increasing number of patients seek dental treatment with the primary motivation of achieving an aesthetic smile. This case demonstrates a patient presenting with an unaesthetic smile associated with multiple carious teeth, gingival recession, and discoloration. We describe how a multidisciplinary approach involving prosthetic, restorative, and surgical disciplines enabled us to achieve an aesthetic smile for the patient. We also highlight the importance of minimally invasive dentistry. Conservative options were preferred to avoid the consequences of the restorative cycles.

## Introduction

In recent years, the demand for aesthetic dental treatments has increased substantially, reflecting patients’ heightened desire for an attractive smile [1]. Designing a smile involves various parameters, including the golden ratio, smile line assessment, incisor proportion and angulation, width-to-height ratios of maxillary anterior teeth, gingival contour, root coverage, and papilla height [2]. Patients may present with multiple factors contributing to an unaesthetic smile, such as skeletal defects, malpositioned teeth, discolored teeth, or uneven gingival margin asymmetry. Tooth discoloration is a common dental concern that presents both clinical and aesthetic challenges. Its characteristics vary according to etiology, appearance, composition, location, severity, and degree of adherence to the tooth surface. Discoloration may arise from extrinsic factors, such as coffee, wine, and tobacco use, or intrinsic causes, including congenital or systemic conditions. Proceeding with treatment without first addressing the underlying etiology may compromise the outcome, as long-term results cannot be achieved [3]. Gingival asymmetry is another aesthetic issue, particularly when discrepancies exceed 2 mm [4].

Treatment options depend on the underlying etiology; tooth discoloration in vital teeth can be addressed with hydrogen peroxide products, while non-vital teeth may benefit from the walking bleach technique. Prosthodontic and restorative approaches may also be employed to enhance aesthetic outcomes, highlighting the importance of a multidisciplinary treatment strategy [5]. Regarding the gingival contour, the gingival zenith plays a crucial role; uneven gingival contour may arise from various etiologies, including iatrogenic factors, gingival recession, gingival enlargement, and other mucogingival conditions [6-8]. In this case report, management of poorly done restorations and correction of gingival recession have been discussed, which explains the multidisciplinary approach to the transformation of an unaesthetic smile to an aesthetic one.

## Case presentation

A 22-year-old male reported to the dental clinic with a chief complaint of an uneven gum surface and discolored front teeth, which made him self-conscious while smiling and affected his self-confidence, and he wanted a smile makeover. The patient revealed a dental history of multiple restorations being done on the affected quadrant, along with the need for constant dental visits due to high caries risk. The patient’s medical history is non-contributory. A seven-day diet recall was noted for the patient. Patient consent was taken to use the photographs of the treatment for publication purposes.

**Figure 1:**
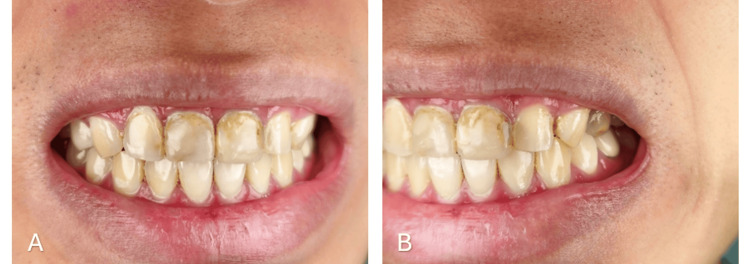
Pre-operative images A: pre-operative unaesthetic smile; B: pre-operative carious maxillary incisors

Upon examination, dental caries was found on the proximal surfaces of 12, 11, 21, and 22, which were poorly restored, aesthetically unappealing, and had uneven labial restorations. Miller’s class 1 gingival recession was seen on 12, along with gingival inflammation on all the upper incisors. The patient revealed that 11 and 21 were root canal-treated, and a periapical radiograph confirmed it (Figures 1A, 1B).

A periodontal examination was performed, and no clinical attachment loss was found on the upper incisors. A thorough professional mechanical plaque removal (PMPR) and polishing was performed, followed by a dietary analysis. The patient was found to consume a diet high in sugar with frequent snacking. Accordingly, dietary advice was given to reduce both sugar intake and snacking frequency, in the hope that further caries development will be reduced. Proximal caries was excavated using a round bur and restored using Brilliant Everglow (COLTENE, Altstätten, Switzerland) composite A2 shade, to be followed by finishing and polishing using a yellow band bur, EVE Diacomp® Plus Twist Polishing Spiral (EVE Ernst Vetter GmbH, Keltern, Germany), along with Diapolisher paste (GC dental, Tokyo, Japan). A comprehensive treatment plan for a smile makeover was proposed and accepted by the patient. This included a prosthetic approach with zirconia crowns for teeth 11 and 21, given the compromised tooth structure following caries excavation; a restorative approach with composite re-restorations for teeth 12 and 22; and a surgical approach involving a coronally advanced flap for tooth 12 to manage the gingival recession. Zirconia was selected over other all-ceramic options, such as lithium disilicate, as the extensively restored teeth presented a higher risk of compromised bonding.

**Figure 2:**
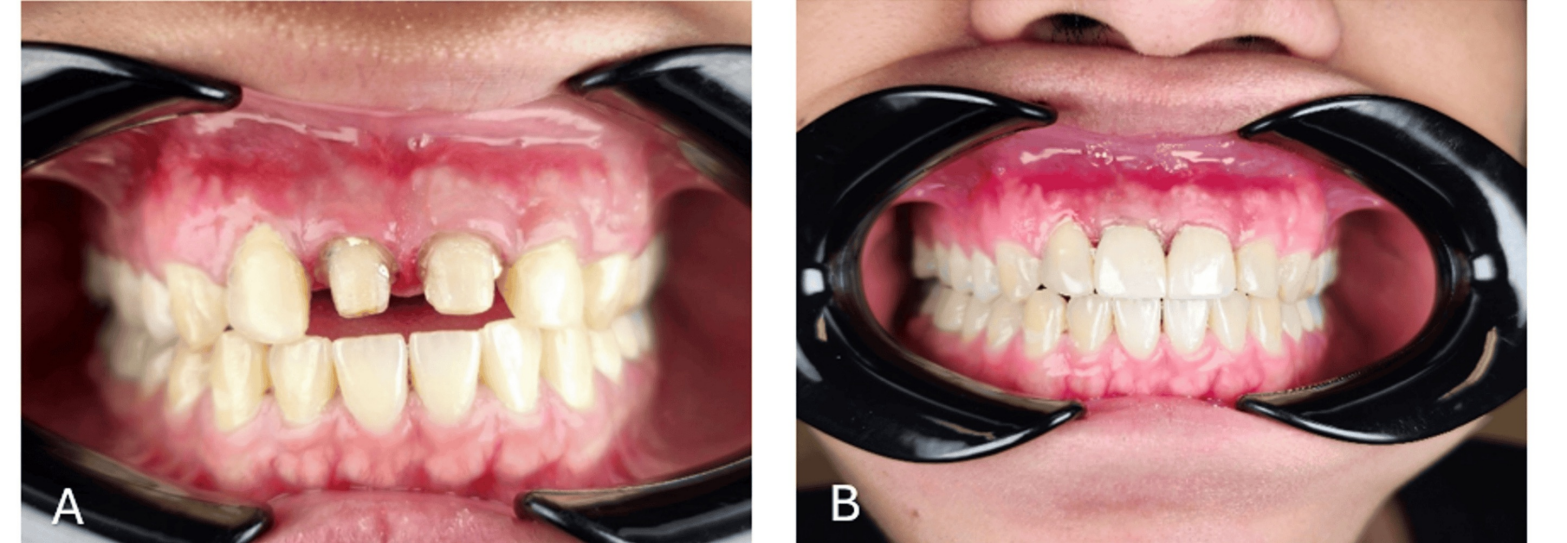
Prosthetic and restorative images A: after crown preparation of 11 and 21; B: completed prosthetic and restorative stage

During the first appointment, an impression and cast were made for the fabrication of a special tray for the patient. In the subsequent appointment, crown preparation was performed on 11 and 21 using a flat-end tapered bur to achieve a shoulder margin (Figure 2A). A subgingival margin was given to avoid the exposure of the finishing line when the patient is smiling. A retraction cord was packed prior to making the impression, which was performed using the multiple-mix technique with light-body and heavy-body addition silicone (3M™ Express™, Solventum, Eagan, Minnesota, USA). A3 shade was selected for the zirconia crown, and zirconia was fabricated for 11 and 21, and cemented using A2 shade resin cement in the final appointment (Figure 2B). Occlusion and proximal contacts were verified.

**Figure 3:**
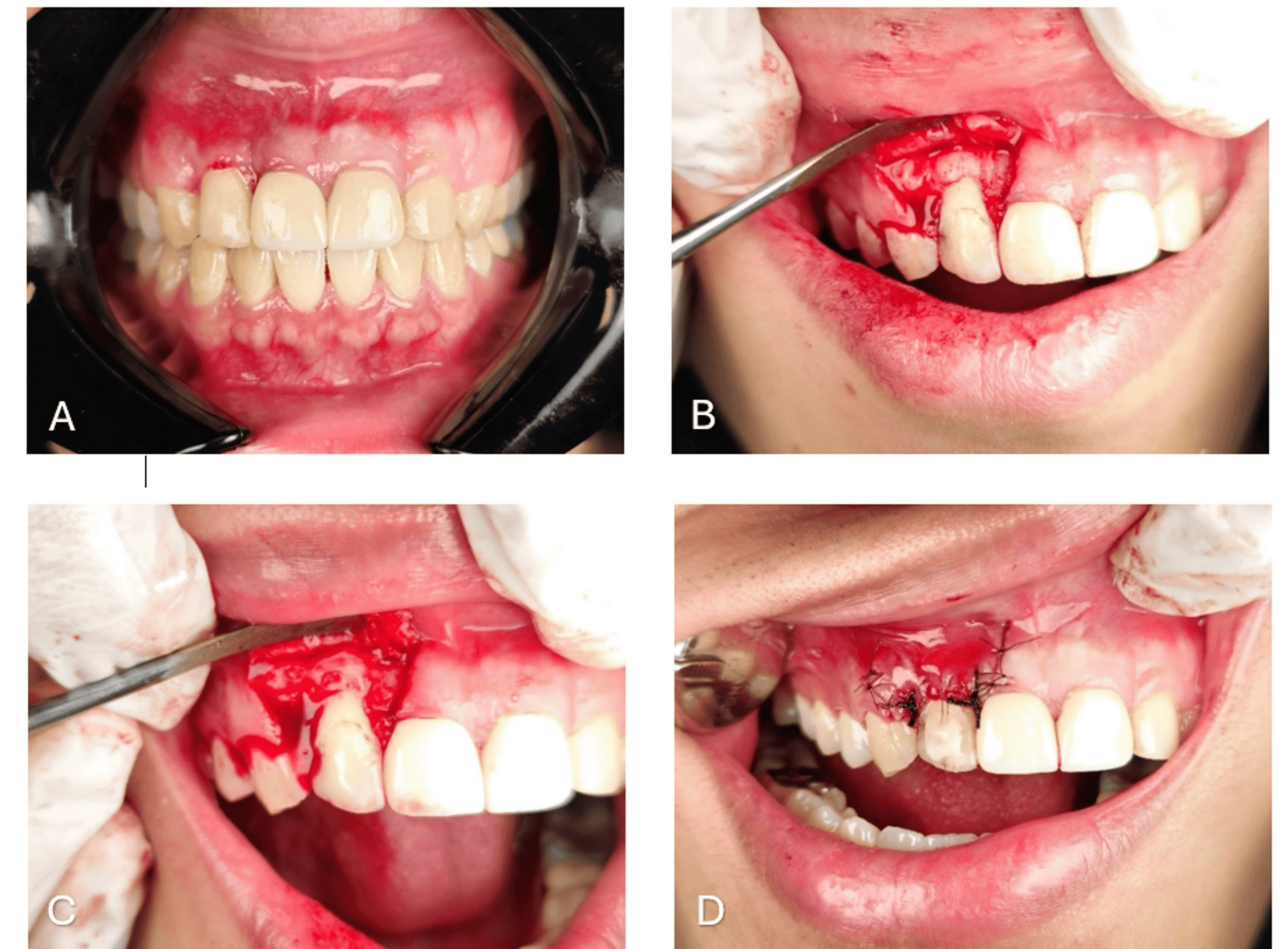
Surgical images A: persisting inflammation on 12; B: bony prominence on 12; C: osteoplasty of bony prominence done; D: sutures placed

Uneven composite on the labial surface of 12 and 22 was smoothened using a polishing bur; restorations were done using Brilliant Everglow A3 body shade composite; finishing and polishing were done using a yellow band bur and EVE Diacomp® Plus Twist Polishing Spiral along with composite polishing paste; and this completed the prosthetic and restorative phase of the treatment plan, and the patient was delighted after seeing the results. Unfortunately, there was still gingival inflammation persisting on 12, along with plaque build-up after repeated non-surgical periodontal therapy despite several reinforced oral hygiene instructions (Figure 3A).

Finally, a decision was made to go forward with the coronally advanced flap despite the presence of inflammation. A papilla preservation flap, along with two vertically releasing incisions extending up to the mucogingival junction, was placed with a Bard Parker 15c blade on 12, and a mucoperiosteal flap was raised using a periosteal elevator. After the flap reflection, a bony prominence was noted on 12 (Figure 3B), following which an osteoplasty was done and the bony prominence was flattened and smoothened using a round bur and bone file, respectively (Figure 3C).

**Figure 4:**
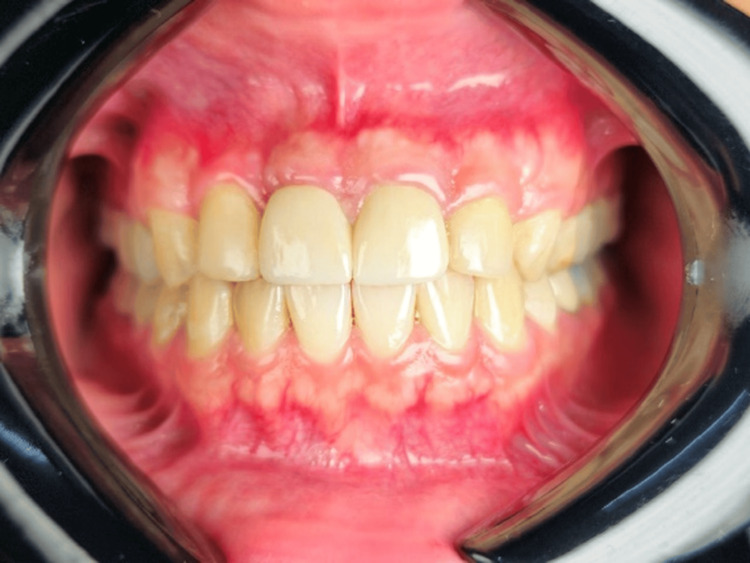
Six months post-operative

**Figure 5:**
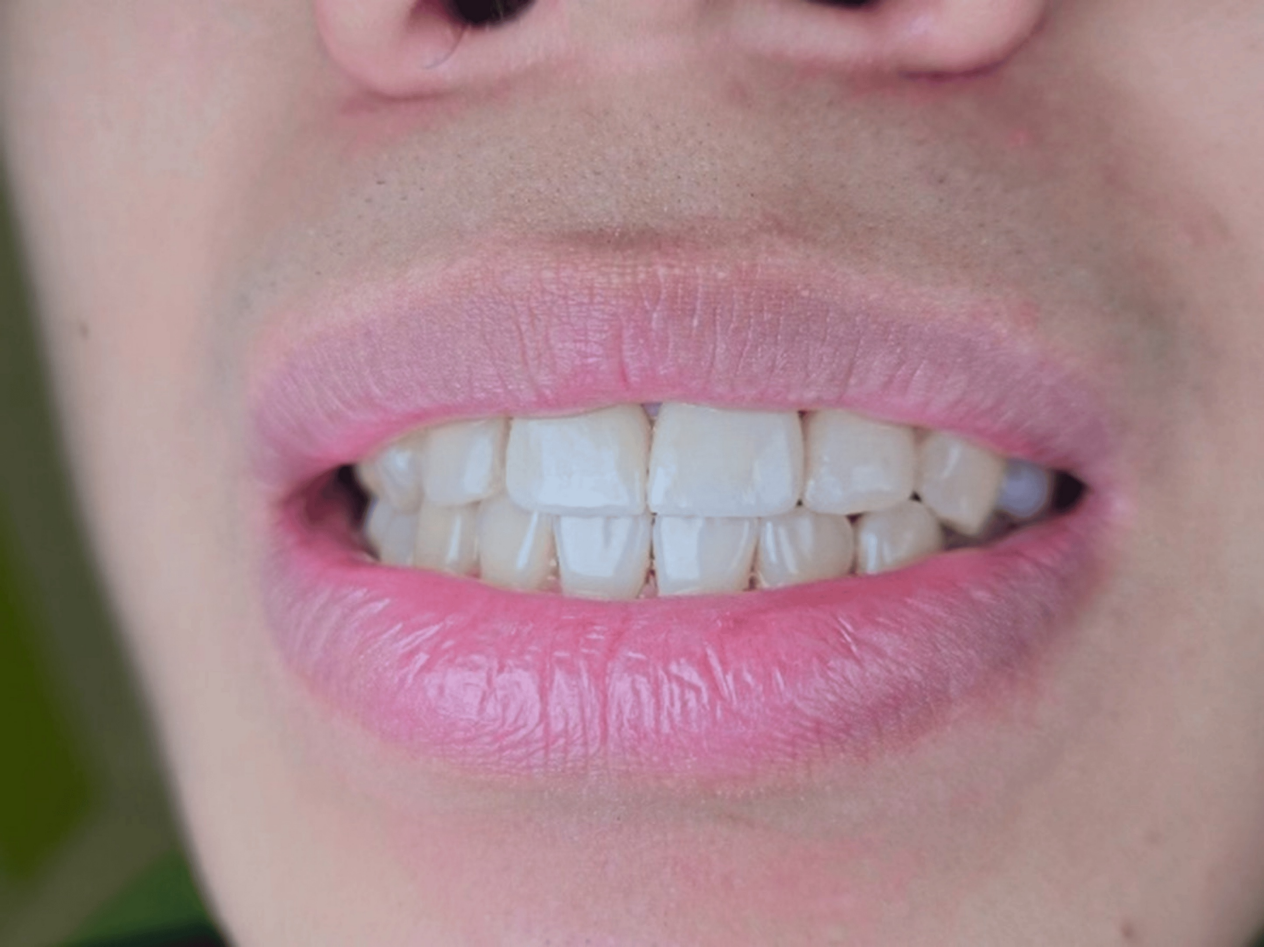
Post-operative smile

Periosteal releasing incisions were given at the base of the flap to advance coronally, sling sutures were placed to stabilize the flap in the advanced position, and simple interrupted sutures were placed for the vertical incision (Figure 3D). The patient was prescribed analgesics and recalled after 10 days for suture removal. A review was done, and healing was uneventful. The patient was recalled after six months for review, and the results were satisfactory. The patient was very happy with the results of the aesthetic smile makeover. Figures 4, 5 show the postoperative smile of the patient without lip retraction.

## Discussion

This case report highlights the importance of a multidisciplinary approach to achieve both an aesthetic and functional outcome for the patient. Multiple parameters were considered, including caries risk assessment, periodontal health, tooth structure integrity, and the aesthetic expectations of the patient.

Periodontal intervention is crucial to improve the gingival symmetry and the smile line of the patient. Although gingivectomy was also a viable management option with low recurrence [9], a coronally advanced flap was ultimately selected. This method was preferred, as it will be able to prevent exposure of the subgingival crown margins of the upper central incisors.

A more conservative option of composite veneer was preferred for the maxillary lateral incisors. Composite veneers were chosen over full coverage crowns to prevent the removal of unnecessary sound tooth structure to avoid the consequences of restorative cycles while achieving an acceptable aesthetic outcome. Every restorative intervention will eventually lead to loss of natural tooth structures, and complications may arise from time to time. Studies have shown that crowning an incisor or canine will lead to an earlier time to the extraction of the restored tooth, as opposed to the placement of a direct restoration [10]. Thus, restoration of life expectancy and a conservative treatment plan were key considerations, which were the deciding factor for the treatment approach to this case [11].

In contrast, full coverage crowns were deemed the most appropriate for the maxillary central incisors (11 and 21) as they were heavily restored with composite. Moreover, to achieve a satisfactory aesthetic outcome, significant tooth structure had to be removed to mask the discoloration, as they were root canal treated, which made full coverage crowns the better option [12]. Studies have shown that the longevity of an all-ceramic crown was expected to be at least five years, and often longer, if they are well maintained [13].

Throughout the treatment, patient expectations and preferences were carefully integrated into the planning process. The patient was mainly concerned about achieving a natural smile while having a desire for a minimally invasive approach if possible. Effective communication with the patient was essential to balance the expectations with clinical feasibility and long-term prognosis.

This case highlights the importance of an individualized treatment plan with a multidisciplinary approach, involving various specialties such as periodontics, restorative dentistry, and prosthodontics. By tailoring interventions based on biological, functional, and aesthetic parameters, clinicians will be able to preserve tooth structure as much as possible and achieve satisfactory aesthetic outcomes. Future follow-up and reviews will be necessary to monitor the longevity of the restoration and periodontal health of the patient over time.

## Conclusions

In dentistry, achieving a balance between function and aesthetics poses significant challenges due to the increased demand and expectations from the patient. A multidisciplinary approach is essential for a comprehensive and conservative treatment plan. This might involve various specialties, including periodontics, restorative dentistry, orthodontics, or even surgery. Formulating a detailed treatment plan before the procedure is crucial. A successful treatment plan would align with the patient’s lifestyle, expectations, and availability. Effective communication is essential to establish mutual understanding and patient commitment. Ultimately, a patient-centered multidisciplinary approach enhances the likelihood of long-term success and satisfaction.
